# The role of consciousness in the phonological loop: hidden in plain sight

**DOI:** 10.3389/fpsyg.2013.00496

**Published:** 2013-08-08

**Authors:** Bradley R. Buchsbaum

**Affiliations:** Department of Psychology, Rotman Research Institute, Baycrest Hospital, University of TorontoToronto, ON, Canada

**Keywords:** working memory, phonological loop, inner ear, inner voice, consciousness

## Abstract

We know from everyday experience that when we need to keep a small amount of verbal information “in mind” for a short period, an effective cognitive strategy is to silently rehearse the words. This basic cognitive strategy has been elegantly codified in Baddeley and colleagues model of verbal working memory, the phonological loop. Here we explore how the intuitive appeal of the phonological loop is grounded in the phenomenological experience of subvocal rehearsal as consisting of an interaction between an “inner voice” and an “inner ear.” We focus particularly on how our intuitions about the phenomenological experience of “inner speech” might constrain or otherwise inform the functional architecture of information processing models of verbal working memory such as the phonological loop; and how, indeed, how ideas about consciousness may offer alternative explanations for the dual nature of inner speech in verbal working memory.

## The role of consciousness in the phonological loop: hidden in plain sight

Working memory is a cognitive system for the maintenance, manipulation, and monitoring of information that is not currently available in the sensory environment. There is extensive empirical evidence showing that working memory is capacity limited: that one can only retain 3 or 4 independent items or objects “in” working memory at a time (Cowan, 2001; Marois and Ivanoff, 2005). But what does it mean for an item—an internal mental representation—to be “in” working memory? A functional or operational definition might say that for something to be in working memory, it must be readily accessible and can be *reported* or otherwise *described* by a subject under study. According to this definition, a way to find out what a person currently holds in working memory is simply to ask them. If we define working memory in this way, that is, as the current contents of memory that are available for subjective report, then we may say that working memory consists only of consciously accessible information.

A key historical precursor to working memory, the Jamesian concept of primary memory, was identified more or less directly with the contents of consciousness. Many modern theorists also see a close connection between working memory and consciousness. For example, Cowan (1993) has proposed that while many mental representations may be in an “activated state” at any given time, only those representations that are within the capacity-limited “focus of attention,” a concept closely related to conscious awareness, are accessible within working memory. Baars (Baars and Franklin, 2003) has argued that consciousness is associated with a limited capacity “global workspace,” akin to working memory, whose focal contents are broadcast to widely distributed specialized networks in the brain.

In the classic working memory model of Baddeley and colleagues (Baddeley and Hitch, 1974; Baddeley, 1992, 2003; Repovs and Baddeley, 2006), however, consciousness is not an explicit motivating force for the logic and structure of the theory. Nevertheless, certain aspects of the model are often informally identified with some characteristics of conscious experience. This is especially clear in the case of the verbal component of working memory, the “phonological loop,” where the resemblance between the model and subjective phenomena seems to be more than merely metaphorical. Our present goal is to show that even a seemingly consciousness-averse information-processing model such as the phonological loop owes something to an introspective analysis of conscious experience. We focus particularly on how our intuitions about the phenomenological experience of “inner speech” might constrain or otherwise inform the functional architecture of information processing models of verbal working memory such as the phonological loop; and how, indeed, the analysis of consciousness may suggest alternative interpretations of the fundamental nature of inner speech in verbal working memory.

## The multi-component working memory model

The goal of the working memory model (Baddeley, 1992) is to provide a basic functional description of how internal mental representations are maintained online during complex cognitive processing. It consists of two so-called “slave systems,” the visuospatial scratchpad and the phonological loop, which are dedicated to the storage of visual and verbal information, respectively. The visuospatial scratchpad and the phonological loop are conceived of as buffers, that is, as containers of highly processed information and are not directly involved in the perceptual analysis of sensory stimuli. Both of these storage subsystems are controlled and monitored by a superordinate cognitive control mechanism called the “central executive.” While the visuospatial scratchpad is described as a single storage component (but see Logie; Logie and Pearson, 1997), the phonological loop consists of two sub-components, a storage component called the phonological store and a maintenance component known as the articulatory rehearsal process. The phonological store can hold speech-based information for a brief period of time (approximately 2 s per item) before it is lost to decay. The role of the articulatory rehearsal process is to counteract this decay by periodically “refreshing” the contents of the phonological store by way of subvocal speech.

## Inner speech as a mnemonic strategy

Because of the importance of language and communication in human cognition, memory for verbal information has been the topic of a great deal of research in the cognitive sciences over the last 50 years. A somewhat trivial (and by now nearly anachronistic) but oft-cited example of the need in everyday life for verbal working memory, is to keep the digits of a phone number “in mind” after reading them from a phonebook or hearing them from a telephone operator. There is a period of time in between receiving the number and dialing it where the ordered sequence of digits must be maintained in working memory; and during this interval most people will “repeat the numbers to themselves,” either overtly or covertly, as a way of keeping the digits conscious and accessible. But what does this behavior, this routine cognitive strategy, tell us about the kind and nature of the internal codes that are used in verbal working memory?

One might ask of course whether subvocal rehearsal is actually beneficial to memory performance. This question has been answered by testing subjects' memory for lists of verbal items while preventing rehearsal by requiring them to concurrently articulate an irrelevant word (e.g., “hiya”) during a delay period interposed between stimulus perception and recall. Many studies have shown that blocking rehearsal through “articulatory suppression” has a strong negative effect on recall performance, suggesting that the cognitive strategy of rehearsal is indeed useful (e.g., Baddeley et al., 1984). A second obvious question is whether for rehearsal to be an effective strategy, the to-be-remembered verbal items must be spoken aloud; if so, it would suggest that rehearsal serves merely as a kind of trick to “re-present” the items to the auditory perceptual system through external sensory feedback loop. In fact, however, studies have shown that verbal rehearsal is beneficial to memory even when it is subvocal and thus produces no external auditory feedback (e.g., Murray, 1968). Here we note that this finding also comports with phenomenological experience: when we “silently talk to ourselves”—when we subvocally rehearse—we seem to *hear* a dim but unmistakable voice; we are *listening* to this voice, and we typically identify this voice as our own. The empirical demonstration that subvocal rehearsal is beneficial to short-term verbal recall, combined with the subjective experience that internal speech involves both an *inner voice* and an *inner ear*, offers intuitive support for the basic architecture of the phonological loop model of verbal working memory, which posits the existence of two such communicating components.

## Sensory and motor codes in the phonological loop

A fundamental aspect of the phonological loop model is that it involves the repeated conversion between two codes: one that is a (quasi-sensory) phonological code and one that is an (quasi-motor) articulatory code (Wilson, 2001). Both of these codes represent verbal content and the transfer from one format to the other does not involve in a net gain or loss of information in the system. Although we have noted that the dual coding premise appeals to our subjective experience of the inner voice and inner ear during covert speech, from an information processing standpoint it seems rather like a pointless game of representational ping-pong. Indeed, Baddeley and Hitch (1974) had initially attempted to explain the main empirical findings of verbal working memory research more parsimoniously in terms of a single articulatory component, without the need for an auditory/perceptual store. This was based on the strong evidence for the critical role of speech production processes in verbal span tasks. For instance, individual differences data showed that the faster a person is able to articulate a set of words, the greater his or her verbal memory span (Landauer, 1962). In addition, sets of words that take longer to articulate result in poorer memory performance than sets of shorter duration words (the word-length effect Baddeley et al., 1975); and, as mentioned previously, blocking subvocal rehearsal through articulatory suppression impairs verbal short-term memory.

Several lines of evidence, however, ultimately compelled the addition of the phonological store component and with it the dual coding view of verbal working memory was established (Salame and Baddeley, 1982). First, neuropsychological investigations showed the existence of patients with dramatically reduced auditory-verbal short-term memory in presence of preserved speech production and auditory comprehension abilities (Shallice and Warrington, 1977; Shallice and Vallar, 1990). Second, articulatory suppression eradicates the phonological similarity effect when verbal stimulus presentation is visual, but not when it is auditory. This finding suggested that the phonological similarity effect was based on an auditory-perceptual code rather than an articulatory one. Third, the ability to make rhyme judgments on a pair of visually presented words is unaffected by articulatory suppression (Baddeley and Lewis, 1981). Fourth, the presentation of irrelevant speech during immediate verbal memory has a deleterious effect on serial recall (Jones and Morris, 1992; Beaman and Jones, 1998), suggesting the existence of a representational code more closely tied to the auditory-sensory system than to the articulatory-motor system.

To account for these data, Baddeley and colleagues split the articulatory loop into an articulatory control process and a phonological store, which act in concert to retain verbal information in working memory (Salame and Baddeley, 1982). In the new model, neither component is on its own capable of supporting maintenance of verbal information in working memory, each has as it were an Achilles heel. The articulatory rehearsal process has no storage capacity of its own, but can *refresh* the contents of the phonological store, which are otherwise subject to rapid time-based decay. The phonological store has a memory capacity of its own, but no internal means of reactivating its decaying contents. Thus, as neither component is self-sufficient, damage to either one of these components should result in severe degradation in the performance of the system. Indeed, the interdependence of two such components is supported by neuropsychological data showing that patients with severe dysarthria, and thus a damaged articulatory control process, have greatly reduced verbal working memory (Baddeley and Wilson, 1985); and, as already mentioned, patients with temporo-parietal lesions have been described with intact speech production and comprehension abilities, but impaired auditory-verbal short-term memory spans.

## The inner ear, the inner voice and the phonological loop

We have briefly reviewed the historical development of the phonological loop and some of the empirical evidence that led to the fractionation of the verbal component of working memory into an articulatory and phonological component. It is interesting to note that the evolution of the phonological loop converged on an architecture that is more compatible with phenomenal experience than its purely articulatory precursor. It may be instructive to consider whether this congruence between introspective evidence and the structure of an information-processing model is more than a coincidence, or whether it may have a deeper significance.

A seemingly arbitrary aspect of the phonological loop is the claim that the articulatory control process has no internal storage capacity. This might translate, in phenomenological terms, to: “the inner voice cannot hear itself speak,” or: “the inner voice is deaf.” If we, for the sake of argument, endow the articulatory control process with storage capacity and the ability to reactivate its own contents (i.e., as in original articulatory loop model), then from an information processing standpoint the component becomes self-sufficient and self-referential: *it is a voice that can hear itself speak*.

Putting aside behavioral considerations for or against such an architecture, it seems to run counter to the introspective evidence telling us that inner speech is a private version of outer speech. Thus, the auditory-perceptual quality of the auditory imagery of the inner ear is *like* hearing external speech, just as when we imagine a patch of green light it is (phenomenologically) *like* seeing a patch of green light (Place, 1956; Smart, 1959; Shepard and Chipman, 1970). Moreover, during inner speech, verbal information constitutes the content of the auditory imagery of the inner ear, and as such is consciously reportable. We cannot say the same for the inner voice: although one can describe a feeling of agency during inner speech (Morsella et al., 2011), this feeling does not carry any linguistic content, and there are no other articulatory-motoric sensations that can be described as representing a verbal message. Thus, an introspective analysis of the phenomenology of inner speech is in favor of the existence of two separable conscious components, and it is not difficult to identify a resemblance between these two phenomena and the functional components of the phonological loop.

## The need for an observer of motor programs

One might say that the inner voice is only identifiable as marker of agency, conveying the feeling that: “it is *you* that is speaking;” whereas the inner ear carries the conscious content of the message: “this is *what* you are saying.” Indeed, the conscious experience of inner actions, including speech production, lack reportable content apart from indicators of agency such as urges, plans, and intentions (Morsella et al., 2011). To enable self-awareness of the content of motor speech programs, such action representations must first be as it were *rendered into sensory-perceptual space*. Thus, we may say that the content of motor programs are not introspectable, they cannot be reflected upon, without first being in some sense realized and observed. This may simply be a necessary property of a self-conscious organism: it cannot anticipate the content of its own actions before these actions have been either explicitly executed or internally simulated (Libet et al., 1982). Another way to understand the impenetrability of the content of motor programs is to assume that neural computations and conscious representations are necessarily independent of one another. In other words, a computational process cannot observe itself: viz. *the inner voice cannot hear itself speak*. We may further note that whereas the computational goal of the action system is to encode motor programs that determine an organism's future interactions with the environment, the primary role of the perceptual system is to decode and represent the content of the sensory world. In this sense, then, the auditory-perceptual system is well suited to perform its regular role as the *observer* in the cortico-cortico crosstalk that is the neural substrate of inner speech (Buchsbaum et al., 2001; Buchsbaum and D'Esposito, 2008).

## The inner ear, the inner voice, and the brain

We have noted a resemblance between subjective experience of inner speech and the two-component structure of the phonological loop. This resemblance may also be seen to extend in to the brain, where even in the 19th century Carl Wernicke referred to the generative process of speech production as consisting of the simultaneous co-activation of “auditory word images,” housed in the superior temporal gyrus, and “motor word images” stored in the inferior frontal gyrus; and they were assumed to be connected by a large fiber bundle spanning across the frontal and temporal lobes called the arcuate fasciculus (Eggert and Wernicke, 1874/1977).

Modern functional neuroimaging studies of inner speech in the context of simple working memory tasks where subjects must keep in mind a small set of words or pseudowords over a delay period have essentially verified Wernicke's hypothesis. Many studies have shown that during subvocal rehearsal robust activation is observed in both frontal “motor” regions (Broca's area, premotor cortex) and posterior “sensory” regions (planum temporale, superior temporal sulcus) that are often implicated in speech perception and production processes (Wise et al., 2001; Hickok et al., 2003; Buchsbaum et al., 2001, 2005, 2011). Indeed, the continuous co-activation of inferior frontal and superior temporal brain sites during inner speech has recently been show to persist for as long as 45 s in a task requiring extended inner speech (Fegen et al., submitted), long after transient executive and cognitive control processes that are activated during stimulus encoding have ceased and the subject has entered an automatic “maintenance mode.” Thus, Wernicke's notion of a simultaneous reverberation between auditory and motor word images, an idea that has an affinity with phenomenological experience of inner speech, finds support from functional neuroimaging studies of subvocal rehearsal.

## Implications for understanding inner speech and verbal working memory

In light of the above discussion, then, one might argue that the “Achilles Heel” of the articulatory rehearsal process is *not*, as is claimed in the phonological loop model, that it lacks *storage capacity*, but rather that it lacks a direct means of delivering information to conscious awareness. Articulatory programs must be routed through the sensory perceptual system to gain access to conscious awareness. Earlier we referred to this aspect of the model as an unnecessary game of representational ping-pong. It is traditionally explained by assuming that the articulatory rehearsal process lacks storage capacity and therefore must continuously access and update representations in the phonological store. However, there is no special reason to assume that the articulatory system lacks storage capacity—in fact, there is reason to think otherwise (e.g., Monsell, 1984; Levelt, 1993). Rather, we propose that the two-component architecture of the phonological loop may be better understood as a emerging from the requirement that articulatory programs must first be *witnessed* by a sensory system before they can gain access to consciousness and working memory. If we take this view, then the concept of a single locus for the temporary storage of phonological information is no longer necessary to explain the inner voice/inner ear duality of verbal working memory. Rather, we may dispense with the notion of temporary storage altogether (e.g., Craik and Kirsner, 1974; Ruchkin et al., 2003; Postle, 2006; Buchsbaum and D'Esposito, 2008), and instead propose that the this duality is a fundamental consequence of the conscious impenetrability articulatory motor programs and the corresponding need for a external representational system into which motor output can be projected. In fact, coordinated activity between anterior “motor” systems and posterior “sensory” systems appears to be a general feature of declarative memory systems across multiple sensory modalities and domains (Danker and Anderson, 2010; Buchsbaum et al., 2012); and thus the literal conversation of inner speech may only be a special case of a neurophysiological principle that dictates that conscious thoughts emerge from the coordinated interplay between anterior and posterior brain systems.

### Conflict of interest statement

The author declares that the research was conducted in the absence of any commercial or financial relationships that could be construed as a potential conflict of interest.
